# Spontaneous Midsubstance Rupture of the Left Little Finger’s Flexor Tendon in the Absence of Trauma

**DOI:** 10.7759/cureus.71164

**Published:** 2024-10-09

**Authors:** Mohd Fareez Othman, Abdul Qayyum Mohd Raziff, Parminder Singh Gill Narin Singh, Shalimar Abdullah

**Affiliations:** 1 Hand and Microsurgery, Fakulti Perubatan, Universiti Kebangsaan Malaysia, Kuala Lumpur, MYS

**Keywords:** little finger, midsubstance rupture, primary tendon repair, spontaneous flexor tendon rupture, tendon reconstructive surgery

## Abstract

Spontaneous midsubstance rupture of the flexor tendon without underlying pathology is rare. This case report describes a healthy 45-year-old man who experienced spontaneous midsubstance flexor tendon rupture at zone III. He presented with pain and disability in flexing his left little finger after carrying a plastic bag containing 20 kg and only sought medical treatment 10 days after the injury. Two-stage tendon reconstructive surgery was performed, involving the insertion of a nasogastric tube in the first stage followed by ipsilateral palmaris longus tendon grafting in the second stage. No underlying pathology was found to cause the tendon rupture. This report also describes the clinical diagnosis and treatment options for flexor tendon injuries, including primary tendon repair and two-stage tendon reconstructive surgery.

## Introduction

An opposing force to an actively flexed digit can cause a closed flexor tendon injury. Based on the mechanism of injury, closed flexor tendon injuries can be classified as traumatic tendon avulsion, attrition rupture, spontaneous midsubstance rupture, infiltrative tenosynovial rupture, and iatrogenic [1]. Tendon avulsion from its insertion is the most common site of flexor tendon injuries, while midsubstance tendon rupture is uncommon, accounting for only about 3.7% of all flexor tendon ruptures [2, 3]. The term 'midsubstance tendon' itself means within the tendon substance. Boyes JH et al., in 1960, described spontaneous tendon ruptures as intratendinous ruptures that occurred without underlying or associated pathological conditions and in the absence of trauma [3]. However, the understanding of spontaneous tendon rupture was still evolving, as Kannus P and Józsa L in 1991 found that pathologic changes, which were mostly degenerative changes and tendinopathy, were detected in all 891 patients with spontaneously ruptured tendons after examining them using light microscopy, electron microscopy, and histochemical techniques [4]. In contrast to the traumatic distal tendon avulsion, which occurs with swelling and inability to flex the affected finger as well as discomfort along the synovial tendon sheath, a patient with midsubstance tendon rupture experiences less dramatic symptoms, only a 'pop' feeling in the palm with infrequent sharp pain [1,2]. This difference in manifestation is important, as a patient with midsubstance flexor tendon rupture may be late in seeking medical attention, and this may change the direction of treatment for flexor tendon injuries, as will be discussed later in this case report. This case report describes a spontaneous midsubstance rupture of the flexor digitorum profundus (FDP) and flexor digitorum superficialis tendons in zone III of the left little finger.

## Case presentation

A 45-year-old healthy gentleman without any medical illnesses complained of pain in his left little finger. The pain started after he rushed while carrying a plastic bag weighing about 20 kg with his little finger. He described it as sharp and non-radiating; however, the symptoms were mild and infrequent, and he did not notice any swelling on his left little finger. The condition worsened when he also could not bend his little finger shortly afterward. It took him 10 days before seeking medical treatment, as initially, he thought it was self-limiting. Upon clinical examination of his left hand in the clinic, a gap was felt at the volar aspect of the proximal phalanx of the left little finger, with disability in the active flexion of the distal interphalangeal joint and proximal interphalangeal joint. However, the active flexion of the metacarpophalangeal joint of the little finger was still present. Otherwise, no swelling, bruises, or wounds were noticed on the little finger. Plain radiographs showed no obvious fracture. No MRI or ultrasound had been performed since MRI was a limited resource and the earliest available date was more than 3 weeks away, while ultrasound was operator-dependent.

**Figure 1:**
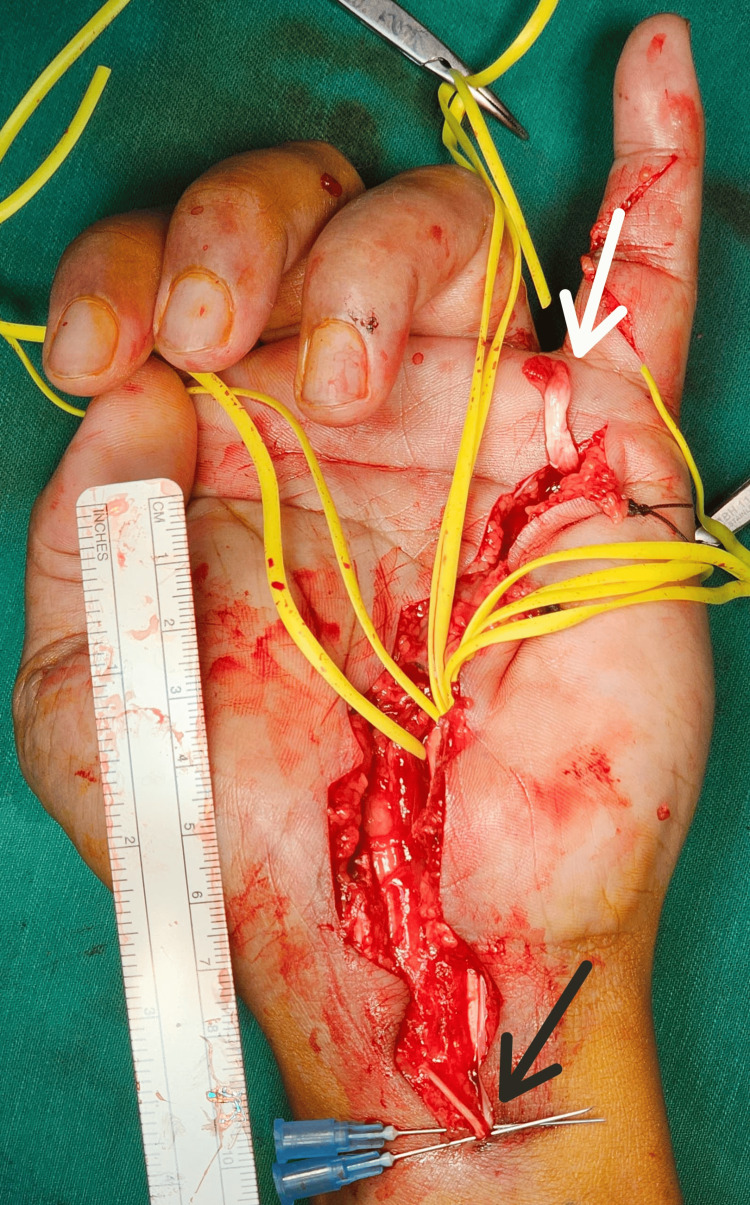
Picture of the ruptured flexor tendon during wound exploration. White Arrow: The distal ruptured tendon stump, found in flexor zone III. Black Arrow: The proximal ruptured tendon stump, found in flexor zone V (proximal to the carpal tunnel), appears macerated.

Hence, he was planned for wound exploration and primary flexor tendon repair, keeping in view a two-stage tendon reconstruction for his left little finger. Intraoperatively, the incision was started at zone II with a Brunner incision, since the gap was felt there during the physical examination. However, the tendon was found intact. The wound exploration was extended to zone III where the distal stump of the ruptured flexor tendon was found. The incision was further extended proximally to the carpal tunnel and to the proximal wrist where the proximal stump of the ruptured tendon was found (zone V). However, the surgeon found the proximal ruptured tendon stump appeared thinned and macerated, which could not be repaired primarily in the surgery (Figure 1).

**Figure 2:**
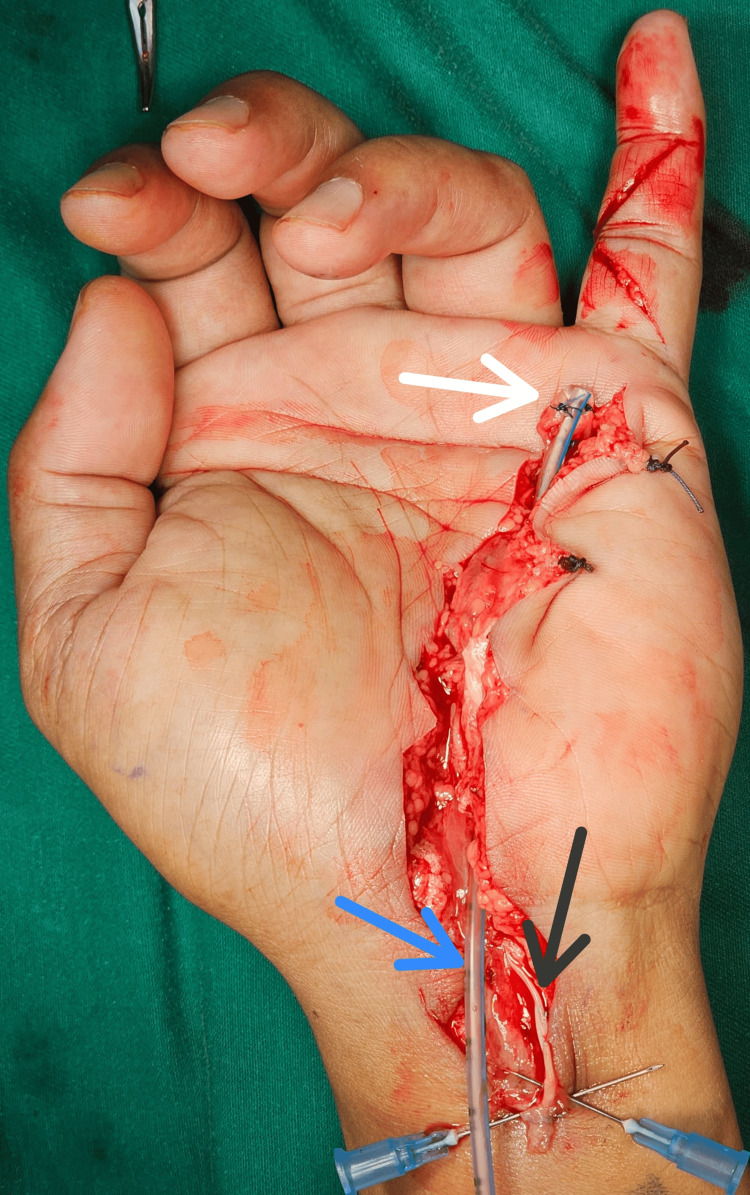
Nasogastric tube insertion during the first stage of tendon reconstruction. White Arrow: The distal tip of the nasogastric tube was sutured to the distal ruptured tendon stump in flexor zone III. Black Arrow: The proximal ruptured tendon stump was sutured to nearby tissue. Blue Arrow: The proximal tip of the nasogastric tube was left free.

Although the patient presented relatively early for primary tendon repair, the poor soft tissue condition led the surgeon to opt for a two-stage tendon reconstruction, allowing a pseudosheath to form first around the inserted nasogastric tube in the first stage. For the first stage, the nasogastric tube was passed through the pulley system and sutured distally to the distal FDP stump. The proximal end of the tube was cut and left freely adjacent to the proximal ruptured tendon stump. The proximal stump of the ruptured tendon was then sutured to the surrounding tissue before the wound was closed (Figure 2).

Postoperatively, the little finger was immobilized for about one week, and he was referred to physiotherapy to achieve full passive flexion until the second stage. During the second stage of tendon reconstructive surgery, which was done one month after the first surgery, the tube was removed, and the ipsilateral palmaris longus tendon was used for tendon grafting. After the two-stage procedure, he was sent to physiotherapy for staged range-of-motion exercises on the left little finger. The patient started passive range of motion exercises two weeks post-operatively and active range of motion exercises four weeks postoperatively. At a 6-month follow-up, the patient was satisfied with the result as he could actively flex his left little finger at the proximal and distal interphalangeal joints to approximately 30-80 degrees and 40-80 degrees respectively, with a power grade of 3 on the Medical Research Council scale. The patient continued physiotherapy for the left little finger to further strengthen muscles and tendons.

## Discussion

Flexor tendon ruptures commonly occur as a result of tendon avulsion at its insertion, while spontaneous midsubstance flexor tendon ruptures are infrequent. This concept of infrequent failure of normal musculotendinous units has been accepted for many years. However, several etiologies have been identified that predispose the midsubstance flexor tendon to rupture or failure. For instance, a study reveals that overuse injuries in animal models can cause alterations in collagen fibers, tendon fibrillation, and tendon thickening, eventually reducing the tissue's ability to resist tensile load [5,6]. In another study, Zbrodowski A et al. postulated the presence of a possible watershed zone between the FDP tendon and the lumbrical muscle origin when they discovered that each lumbrical muscle receives its arterial supply from four sources but has no anastomoses found between the networks supplying the lumbricals and the FDP tendon at the palm [7].

According to Bois AJ et al. 2007, the most common site for spontaneous flexor tendon ruptures is Zone III (80%), followed by Zones II (14%), and IV (6%) [2]. The different zones of tendon rupture raise questions about the need for imaging, especially ultrasonography and MRI. Ultrasound and MRI are useful for preoperative diagnosis, particularly in evaluating equivocal flexor tendon injuries and enabling the surgeon to locate the site of tendon rupture [8]. In fact, by having preoperative imaging, we can avoid excessive surgical approaches by providing a more conclusive diagnosis and supporting the clinical examination [9]. However, no MRI or ultrasound was performed in this case because MRI was a limited resource and the earliest available date was more than 3 weeks away, while the ultrasound was operator-dependent.

The amount of time between the rupture and the therapy, the location of the rupture, and the state of the torn tendon ends are crucial considerations in the decision-making process for surgical treatment. Suppose the ruptured tendon ends are not significantly frayed or attenuated; patients presenting during the first three weeks of the injury can be treated with primary tendon repair, as primary surgical repair has a better functional outcome than secondary tendon repair or tendon graft surgery [10].

For every tendon repair or tendon graft, the surgeon needs to bear in mind that excessively tight repair may cause the quadriga effect. The quadriga effect is characterized by a flexion lag in the finger next to the one with a shortened FDP tendon, due to shared muscle bellies of the FDP tendon. The FDP muscle belly, shared by the long, ring, and little fingers, can cause the quadriga effect even with a 1 cm reduction [11]. Therefore, over-tensioning of the repaired tendon needs to be avoided intraoperatively.

Other than primary tendon repair, interposition tendon grafts or tendon transfers are also appropriate treatments for ruptures in zone III in patients who present more than three weeks following the injury [12]. In this case, even though the presentation is less than three weeks, the condition of the tendon is frayed; hence, two-stage tendon reconstruction surgery is chosen.

The soft tissues must be prepared for the final implantation of a tendon graft once the decision is made to proceed with a two-stage tendon reconstruction. A temporary nasogastric tube is inserted, the pulley and sheath system are rebuilt, and supervised rehabilitation is performed to regain as much flexibility as possible before the second stage of surgery. Stage 1 involves inserting a gliding implant into the damaged tendon bed. This implant will create a pseudosheath around the rod that is coated with mesothelial cells, lubricating it like synovial fluid. Stage 2 involves inserting a tendon graft into this pseudosheath [12].

In certain zones, particularly zones III, IV, and V, the sheath and pulley system do not need to be reconstructed, due to their location, which is proximal to the tendon sheath. However, because the ruptured tendon is macerated, staged reconstruction surgery is best for this case. In fact, patients who have a soft tissue defect, an infection, or segmental loss may require a delayed form of reconstruction [13].

Staged reconstruction may have complications after surgery. The most frequent side effects are adhesions, which can appear along the graft's surface or at graft junction sites [14]. However, proper surgical technique and an effective postoperative therapy regimen may reduce the formation of adhesions. Other complications of surgery include graft ruptures that may occur due to poor surgical technique as well as excessively aggressive rehabilitation; this may happen at both proximal and distal graft junctures [15].

Besides the quadriga effect, as previously discussed, loose graft tension may cause lumbrical-plus finger [16]. This condition occurs when lumbrical muscles pull, causing hyperextension of the interphalangeal joint of the affected fingers during fist formation. Finally, complications of surgery include joint contracture and infection.

## Conclusions

In conclusion, spontaneous midsubstance rupture of the flexor tendon is rare. The treatment depends on several important factors, including the time of presentation after the injury as well as the soft tissue condition of the ruptured tendon. A surgeon should consider primary tendon repair if the presentation is less than three weeks and the soft tissue condition is good. However, if the presentation is more than three weeks and the soft tissue condition is poor, the surgeon may consider other alternatives, such as two-stage tendon reconstruction, as described by this case report. Last but not least, preoperative imaging such as MRI and ultrasound will be helpful in managing close flexor tendon injuries.
